# From osmotic imbalance to germination arrest: key physiological nodes in the drought-alkaline interaction of alfalfa

**DOI:** 10.3389/fpls.2025.1670504

**Published:** 2025-10-31

**Authors:** Yunfei Zhang, Hongtao Hang, Xiaomei Wan, Guoling Guo, Chuanting Li

**Affiliations:** ^1^ School of Karst Science, Guizhou Normal University, Guiyang, China; ^2^ State Engineering Technology Institute for Karst Desertification Control, Guiyang, China; ^3^ State Key Laboratory of Environmental Geochemistry, Institute of Geochemistry, Chinese Academy of Sciences, Guiyang, China

**Keywords:** alfalfa, drought stress, bicarbonate stress, drought-salt combined stress effect, germination physiological response

## Abstract

Alfalfa (*Medicago sativa* L.) is often constrained by factors such as drought and salinity-alkalinity in cultivation. This study aims to investigate the differential response characteristics and physiological-ecological mechanisms of alfalfa during the germination stage under drought, bicarbonate, and drought-salt combined stress. Drought stress was simulated using PEG-6000 (0–20%), and salinity-alkalinity stress was simulated using NaHCO_3_ (0–30 mM) to conduct germination tests on 12 alfalfa varieties. Based on the semi-inhibitory concentrations of germination and phenotypic indicators of the 12 alfalfa varieties under either drought or bicarbonate stress, drought-salt combined stress was applied. A membership function method was used to comprehensively evaluate the differential responses of the 12 alfalfa varieties to drought and salinity during germination, as well as the physiological-ecological response mechanisms of drought-tolerant, salt-tolerant, and drought-salt sensitive varieties. (1) There were significant differences among varieties in response to single drought or bicarbonate, identifying drought-tolerant varieties WL440HQ and WL363HQ, salt-tolerant variety WL525HQ, drought-sensitive 30°N, and salt-sensitive WL343HQ. Comprehensive evaluation indicated that the strongest and weakest drought and salt-resistant varieties were WL363HQ and WL319HQ, respectively; (2) Drought-salt combined stress exhibited antagonism at low concentrations and synergism at high concentrations; (3) The drought-salt tolerant variety WL363HQ adapted well to the combined stress through mechanisms involving SOD activity and soluble sugars, while the sensitive variety WL319HQ performed poorly. This study provides a scientific basis for elucidating the germination response mechanisms of plants under drought-salt combined stress and offers scientific support for planting in arid and saline-alkaline regions based on the selected drought- and salt-tolerant varieties.

## Introduction

Alfalfa (Medicago sativa L.), as the most widely cultivated leguminous forage grass in the world, is renowned as the ‘King of Forages.’ It is characterized by high protein content, tolerance to cutting, strong regenerative ability, adaptability, and ecological restoration functions (Klabi et al., 2018), It also shows great potential in the field of biomass energy. Studies have shown that the stems of alfalfa are rich in cellulose and hemicellulose, with a theoretical ethanol yield of about 150 liters per ton of dry matter, making it a highly promising raw material for cellulosic ethanol (Lamb et al., 2007; Warnke and Ruhland, 2016). In addition, alfalfa has biological nitrogen fixation capability, which can significantly reduce the use of nitrogen fertilizers, lower life-cycle greenhouse gas emissions, and enhance the sustainability of energy production (Filippa et al., 2020). Its perennial nature and deep root system also provide good stress resistance and adaptability to marginal lands, offering an important species foundation for developing non-food biomass energy in arid and semi-arid regions (Kerckhoffs and Renquist, 2013). However, global climate change has intensified the co-occurrence of soil salinity and drought stress, especially in ecologically vulnerable areas such as Northwest China, the Yellow River Delta, and karst regions. These stresses have become major constraints on agricultural productivity and ecological restoration efforts.

Statistically, China accounts for nearly 10% of the world’s saline-alkaline land, with approximately 7.66 × 10^6^ hectares located in arid and semi-arid zones alone. This highlights an urgent need for the selection, breeding, and promotion of stress-tolerant crops (Guo et al., 2023; Zhu et al., 2024). Karst regions, characterized by unique geological features, climate conditions, and geochemical processes, have developed distinct ecosystems marked by challenges such as karst drought, high pH, elevated calcium and bicarbonate levels, and low nutrient availability (Liu et al., 2021a). Therefore, understanding the response mechanisms of alfalfa to drought and bicarbonate and identifying tolerant varieties are of great significance for ensuring food security and restoring fragile ecosystems.

Drought and saline stress frequently occur together and synergistically inhibit plant growth through osmotic stress, ion toxicity, and oxidative damage (Angon et al., 2022). High concentrations of bicarbonate (HCO_3_^-^) in saline soils not only destabilize cell membranes but also interfere with osmotic regulation. Bicarbonate leads to chlorophyll degradation and impaired photosynthesis (Fang et al., 2021), while drought exacerbates imbalances in water and nutrient uptake. Studies indicate that tolerance mechanisms effective under single-stress conditions may fail or even produce antagonistic effects under compound stress. For instance, salt-induced proline accumulation may be reduced under drought due to conflicting energy allocation, thereby weakening plant resistance (Liu et al., 2021b; Zhu et al., 2022; Li et al., 2003). Moreover, HCO_3_^-^, a typical component of saline soils, exerts a distinct stress mechanism compared to NaCl and requires specific physiological adaptations—such as regulation of carbonic anhydrase activity and hormone signaling—to mitigate ionic toxicity (Ma et al., 2022; Zhang et al., 2024). Despite this, most existing studies focus on individual stressors, with limited systematic exploration of alfalfa’s response patterns under combined stress or criteria for variety selection.

In recent years, significant progress has been made in understanding the physiological and molecular mechanisms of stress tolerance in alfalfa, yet most studies have focused on single stress factors, with scattered and variably weighted indicators, making it difficult to reflect the combined stresses experienced in the field. A multi-factor evaluation system can better simulate complex stress environments closer to natural conditions, while simultaneously considering the comprehensive physiological and growth responses of plants under multiple stress conditions, revealing potential synergistic or antagonistic interactions between different stress factors (Abdelraheem et al., 2021; He et al., 2025). This is crucial for a comprehensive understanding of plant stress tolerance mechanisms. For example, studies have found that L-arginine can mitigate the inhibitory effects of drought stress on maize seedlings by enhancing photosynthesis and antioxidant enzyme activity (Sun et al., 2023). Under salt stress, high salinity disrupts plant ion homeostasis and osmotic balance, affecting physiological processes such as photosynthesis (Guan et al., 2020; Peng et al., 2023). Multi-factor evaluation systems can identify excellent germplasm with broad-spectrum tolerance to multiple stresses, This is particularly important for developing highly resistant crop varieties in the context of climate change, which has led to increasingly frequent extreme weather events, such as prolonged droughts and soil salinization (Abdelraheem et al., 2021; Wang et al., 2023). For instance, in assessing the salt tolerance of alfalfa, a multi-indicator fuzzy comprehensive evaluation model based on LiDAR and hyperspectral image data enabled rapid, non-destructive, and automated screening for salt tolerance (Zhang et al., 2023). Furthermore, the use of genetic engineering to enhance plant stress tolerance also relies on understanding the mechanisms of response to multiple stresses. For example, overexpression of the GsZFP1 gene from soybean can simultaneously improve drought and salt tolerance in transgenic alfalfa, while co-transformation with the CsLEA gene has been shown to synergistically enhance tolerance to both drought and salt stress (Tang et al., 2013; Zhang et al., 2016). Overexpression of the Arabidopsis H^+-PPase gene AVP1 significantly increases the tolerance of transgenic alfalfa to 200 mM NaCl and water deficit (Bao, 2009).

The lag in research on compound stress primarily stems from the complexity of their interaction effects. For instance, HCO_3_^-^ in saline soils can affect root water uptake by modulating pH, while drought intensifies ion enrichment in the rhizosphere, creating a “low water–high salt” vicious cycle (Custos et al., 2020). Recently, Wu Yanyou’s team introduced the concepts of “bicarbonate utilization capacity” and the “water-energy coupling model” in selecting suitable plants for karst environments, offering new insights into compound stress mechanisms (Li et al., 2023; Xia and Wu, 2022; Zhao et al., 2023). Additionally, An Yuan’s group found that circadian regulation of flavonoid metabolism in alfalfa can synergistically alleviate both salt and drought stress, highlighting the importance of time-dynamic analyses (Su et al., 2025). Nevertheless, current studies lack a systematic screening framework from single-factor to compound stress, and there remains no consensus on the weighting of morphological and physiological indicators.

To address these gaps, this study selected twelve alfalfa varieties and simulated drought stress (0–20% PEG-6000) and bicarbonate stress (0–30 mM NaHCO_3_). We analyzed the differential responses of these varieties to single drought or bicarbonate stress and clustered them based on multiple germination, growth, and biomass indicators using the subordinate function method. Furthermore, we explored the physiological mechanisms of drought-tolerant/salt-sensitive and drought-sensitive types under combined stress. By constructing a hierarchical evaluation framework of “single-factor screening – compound stress validation,” this study provides a scientific basis for efficient and resilient pasture breeding, fills a critical gap in alfalfa drought-salt compound stress resistance evaluation, and offers theoretical support for optimizing pasture species deployment in ecologically fragile karst regions.

## Materials and methods

### Plant materials

The alfalfa seeds used in the experiment were obtained from Beijing Zhengdao Ecological Science and Technology Co., Ltd. Detailed information on the twelve alfalfa varieties is presented in **Table 1**.

**Table 1:** Information on the twelve Alfalfa varieties.

No.	Variety name	Thousand grain weight(g)	Germination rate(%)	Place of origin
1	WL363HQ	2.15	90.00	USA
2	WL525HQ	2.64	85.00	USA
3	WL168HQ	2.24	95.83	CA
4	WL298HQ	2.23	96.67	USA
5	WL712	2.94	83.33	USA
6	WL440HQ	2.49	83.33	USA
7	WL358HQ	2.21	86.67	USA
8	WL319HQ	2.00	89.17	USA
9	WL343HQ	2.23	89.17	CA
10	30 N°	2.32	88.33	USA
11	Platu	2.10	83.33	DE
12	WL354HQ	2.41	93.33	USA

### Experimental design

The experiment was conducted in an artificial climate chamber at the laboratory of the Karst Research Institute, Guizhou Normal University. Healthy and plump seeds from twelve alfalfa varieties were selected, surface-sterilized with 1% (v/v) sodium hypochlorite solution for 15 minutes, rinsed three times with distilled water, and placed on double-layer qualitative filter paper in Petri dishes. The dishes were then transferred to the artificial climate chamber for germination under controlled conditions: a 12-hour light/12-hour dark photoperiod, light intensity of 5,500 lx, temperature maintained at 25 ± 1°C, and relative humidity of 50%.

1. Single drought stress: Solutions containing 5%, 10%, 15%, and 20% (w/v) PEG-6000 were prepared using distilled water as the control (CK, 0%). Among these, 0–10% PEG-6000 represented low drought stress, 10–15% medium drought stress, and 15–20% high drought stress. PEG-6000 is a commonly used and controllable drought simulation material that induces plant dehydration by lowering water potential. Its effects are similar to soil drought but not identical.
2. Single bicarbonate stress: NaHCO_3_ solutions at concentrations of 5 mM, 10 mM, 15 mM, 20 mM, 25 mM, and 30 mM were prepared using distilled water, with distilled water serving as the control (CK, 0 mM). Accordingly, 0–10 mM NaHCO_3_ was classified as low bicarbonate stress, 10–20 mM as medium bicarbonate stress, and 20–30 mM as high bicarbonate stress.
3. Drought-salt composite stress: Based on the results from the single drought and bicarbonate stress treatments, 10% PEG-6000 and 15 mM NaHCO_3_ were selected as baseline levels for simulating composite stress. The combined treatment combinations are detailed in **Table 2**. Two representative varieties—drought- and salt-tolerant WL363HQ and drought- and salt-sensitive WL319HQ—were selected for further analysis under composite stress conditions.

**Table 2:** Concentration ratio of the compound stress treatment solution.

Group	No.	PEG-6000 concentration (m/v, %)	NaHCO_3_ concentration (mM)
Group A Compound stress	A1	10	0
	A2	10	10
	A3	10	15
	A4	10	20
	A5	10	25
	A6	10	30
Group B Compound stress	B1	0	15
	B2	5	15
	B3	10	15
	B4	15	15
	B5	20	15

A3=B3 in the table.

### Measurement indicators and methods

According to the “Grass Seed Inspection Protocol: Germination Test” (National Standardization Administration of China, 2016), the number of germinated seeds was recorded daily from sowing until the end of the 10-day germination test (germination defined as radicle emergence ≥ 2mm). On the 10th day, nine uniformly grown seedlings were selected from each treatment for measurement of phenotypic indices, including shoot length and root length. Subsequently, the fresh weight of each seedling was measured, followed by killing the samples in an oven at 105°C for 30 minutes, and then drying at 85°C for 48 hours to constant weight to determine dry weight. The following formulas were used to calculate the germination-related indices:

(1) Germination Rate (GP, %) = (Number of germinated seeds/Total number of tested seeds) × 100

(2) Germination Potential (GE, %) = (Number of germinated seeds within 4 days/Total number of tested seeds) × 100

(3) Germination Index (GI) = ∑(GT/DT)

  Where GT is the number of germinated seeds on day T; DT is the corresponding germination time (in days)

(4) Vigor Index (VI) = GI × S

  Where GI is the germination index; S is the root length (mm)

(5) Relative Value = Experimental Group/Control Group

  Relative value indices included relative germination rate (RGP), relative germination potential (RGE), relative germination index (GI), relative vigor index (RVI), relative shoot length (RSL), relative root length (RRL), relative fresh biomass (RFB), and relative dry biomass (RDB).

(6) Relative Water Content (%) = [(Fresh Weight – Dry Weight)/Fresh Weight] × 100

For physiological measurements, reference was made to *Principles and Techniques of Plant Physiology and Biochemistry Experiments (Li et al., 2000). On day 10, whole seedlings were collected. Malondialdehyde (MDA) and soluble sugar (SS) contents were determined using the thiobarbituric acid (TBA) colorimetric method. Superoxide dismutase (SOD) activity was assessed using the nitroblue tetrazolium (NBT) photochemical reduction method. All measurements were performed with three biological replicates per index.

### Data analysis and comprehensive evaluation

Data processing was performed using Microsoft Excel 2019, while statistical analyses—including one-way ANOVA, two-way ANOVA, cluster analysis, correlation analysis, and regression analysis—were conducted using SPSS 27.0 software (SPSS Inc., Chicago, USA). All results were presented as mean ± standard deviation.

To comprehensively evaluate the drought and salt tolerance of germinating seeds from twelve alfalfa varieties, the affiliation function method was applied (Guo et al., 2019). Under varying concentrations of PEG and NaHCO_3_ stress, the relative germination rate, relative germination potential, relative germination index, relative vigor index, and relative water content of each variety were calculated. Based on these values, the affiliation function scores were computed and averaged with assigned weights. Subsequently, SPSS software was used for cluster analysis and comprehensive ranking of the twelve alfalfa varieties. The formulas used in the affiliation function method are as follows:

(1) If the j-th indicator is positively correlated with stress tolerance:


$${\text{Y}}_{\text{ij}}=({\text{X}}_{\text{ij}}-{\text{X}}_{\text{j min}})/({\text{X}}_{\text{j max}}-{\text{X}}_{\text{j min}})$$

(2) If the j-th indicator is negatively correlated with stress tolerance:


$${\text{Y}}_{\text{ij}}=1-({\text{X}}_{\text{ij}}-{\text{X}}_{\text{j min}})/({\text{X}}_{\text{j max}}-{\text{X}}_{\text{j min}})$$

Where:

${\text{Y}}_{\text{ij}}$ : Affiliation function value of the j-th indicator for the i-th variety.

${\text{X}}_{\text{ij}}$ : Mean value of the j-th indicator for the i-th variety.

${\text{X}}_{\text{j min}}$ : Minimum mean value of the j-th indicator across all varieties.

${\text{X}}_{\text{j max}}$ : Maximum mean value of the j-th indicator across all varieties.

## Results and analyses

### Effect of drought stress on alfalfa seed germination

Most germination and phenotypic indices of the twelve alfalfa varieties were significantly influenced by variety, drought stress level, and their interaction. Fresh and dry weights were highly significantly affected by both variety and drought stress (P < 0.01), but not by their interaction. Relative water content did not show significant variation among varieties; however, it was significantly affected by drought stress and the interaction between variety and drought stress (P < 0.01; **Table 3**).

**Table 3:** Two-way ANOVA of the effects of drought stress and variety and their interaction on alfalfa germination.

Factor		Variety		Drought stress		Variety×Drought stress	
		F	P	F	P	F	P
Germination indicators	Germination potential	62.553	**	3617.27	**	24.467	**
	Germination rate	43.837	**	2667.472	**	21.102	**
	Germination index	364.897	**	18374.171	**	72.91	**
	Vitality index	29.417	**	300.694	**	11.291	**
Phenotypic indicators	Germ length	3.472	**	246.563	**	2.012	**
	Radicle length	25.22	**	108.778	**	9.699	**
	Radicle length/germ length	14.969	**	13.008	**	5.637	**
	Fresh weight	4.501	**	191.073	**	1.578	ns
	Dry weight	4.368	**	5.32	**	1.502	ns
	Relative water content	2.541	ns	274.103	**	2.13	**

**indicates a significant extreme difference (P<0.01), and ns indicates no significant difference (P>0.05).

**Figure 1:**
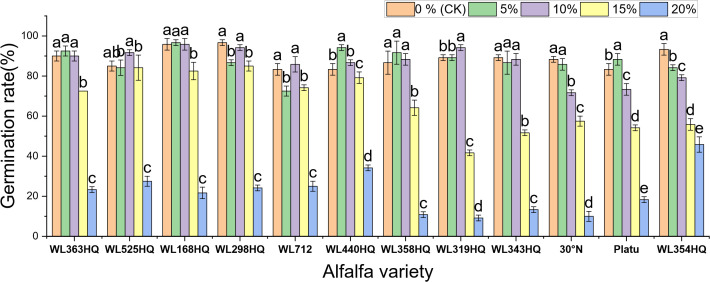
Germination rate of alfalfa seeds under drought stress. Different lowercase letters indicate significant differences (P<0.05) in the same index of the same variety of alfalfa under drought stress at different concentrations of PEG, and % indicates different mass concentrations of PEG. The vertical axis represents different varieties of alfalfa. The same applies hereinafter.

**Figure 2:**
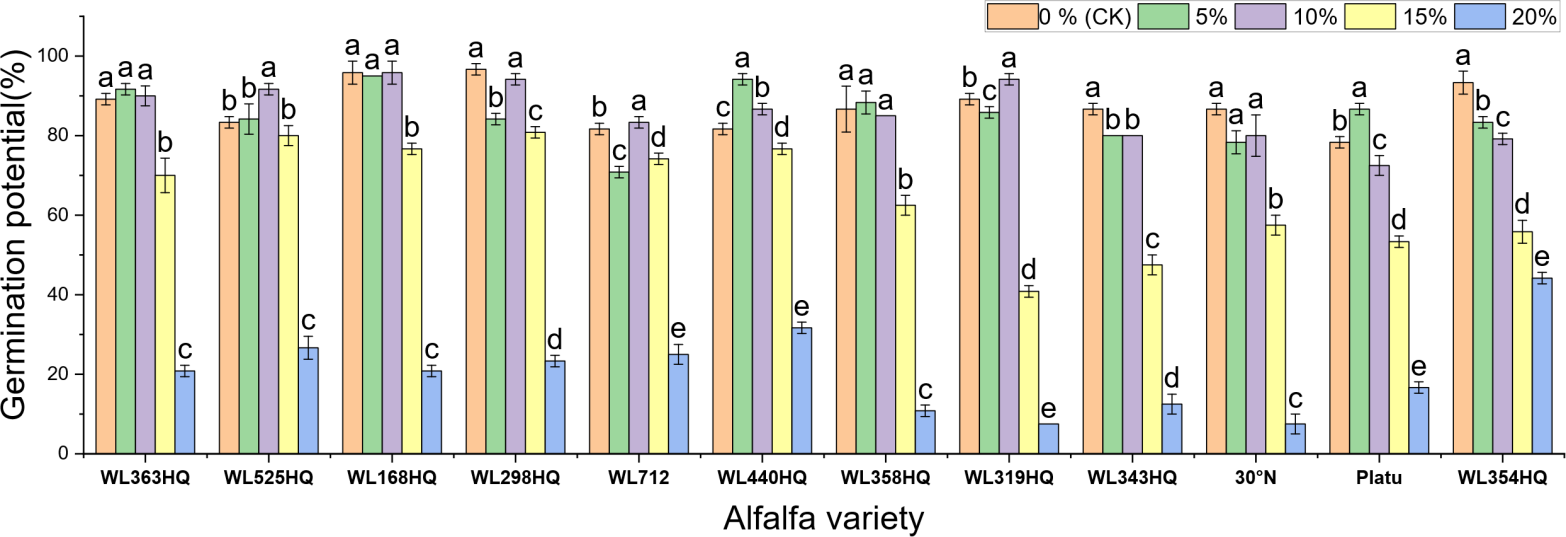
Germination potential of alfalfa seeds under drought stress. Different lowercase letters indicate significant differences (P<0.05) in the same index of the same variety of alfalfa under drought stress at different concentrations of PEG, and % indicates different mass concentrations of PEG. The vertical axis represents different varieties of alfalfa. The same applies hereinafter.

**Figure 3:**
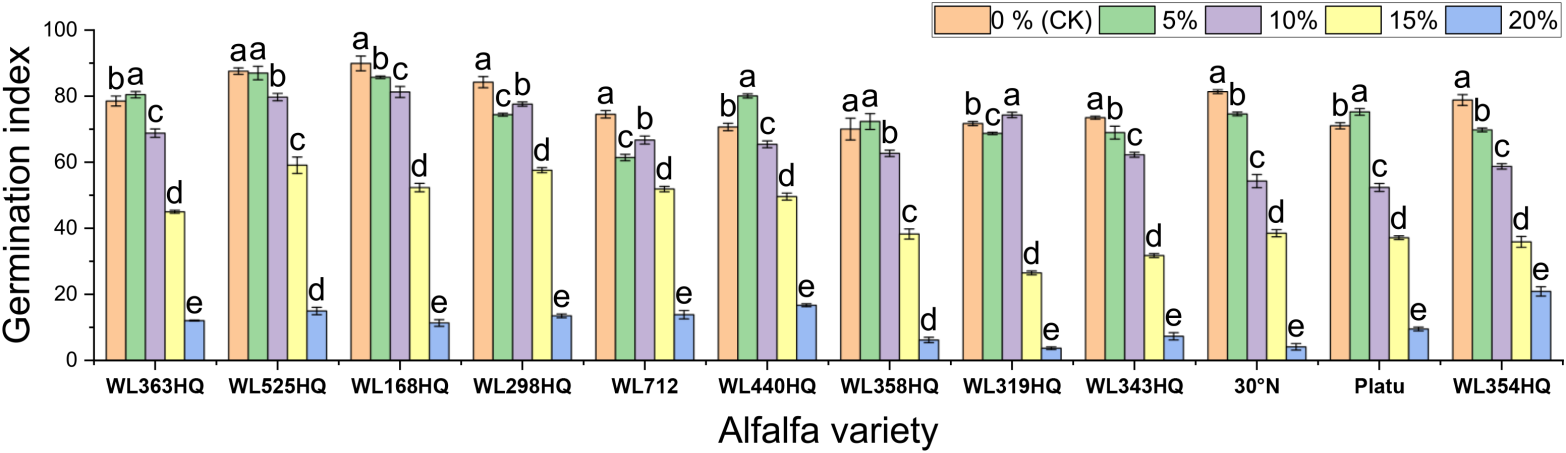
Germination index of alfalfa seeds under drought stress. Different lowercase letters indicate significant differences (P<0.05) in the same index of the same variety of alfalfa under drought stress at different concentrations of PEG, and % indicates different mass concentrations of PEG. The vertical axis represents different varieties of alfalfa. The same applies hereinafter.

**Figure 4:**
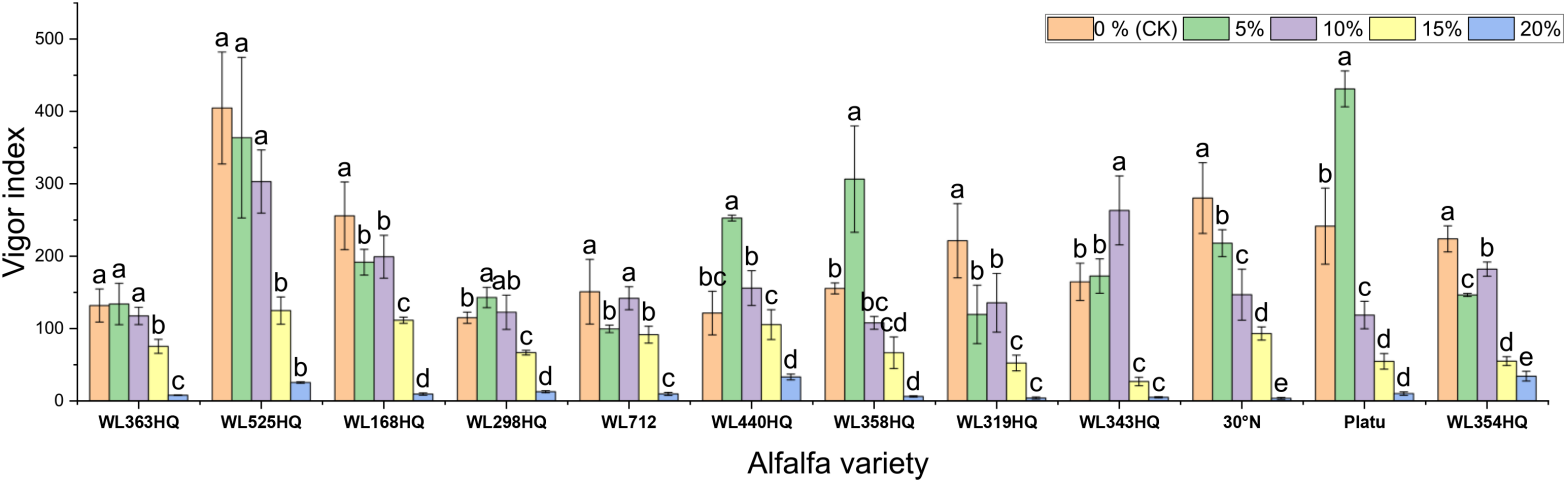
Vigor index of alfalfa seeds under drought stress. Different lowercase letters indicate significant differences (P<0.05) in the same index of the same variety of alfalfa under drought stress at different concentrations of PEG, and % indicates different mass concentrations of PEG. The vertical axis represents different varieties of alfalfa. The same applies hereinafter.

**Figures 1**–**4** illustrate the changes in germination indices of the twelve alfalfa varieties under drought stress. The germination rates were significantly affected by both variety and PEG-6000 concentration (P < 0.01; **Table 3**). As PEG concentration increased, the germination rates of ten out of the twelve varieties remained stable or slightly increased at low concentrations and then declined at higher concentrations. In contrast, the germination rates of 30°N and WL354HQ continuously decreased with increasing PEG levels.

Compared to the control group, Platu exhibited the highest germination rate at 5% PEG concentration. At 10% PEG, WL363HQ, WL168HQ, WL440HQ, WL358HQ, WL319HQ, and WL343HQ showed relatively high germination rates. However, a significant decline occurred beyond 10% PEG. Among them, WL168HQ achieved the highest germination rate of 95.83%, nearly equal to that of the control. WL525HQ maintained a high germination rate of 84.17% even at 15% PEG, although it dropped significantly beyond this level (P < 0.05). At 20% PEG, all varieties showed significantly reduced germination rates ranging from 50.89% to 89.72%. WL354HQ had the highest germination rate (45.83%), while WL319HQ had the lowest (9.17%). These results suggest that 20% PEG may represent or approach the critical threshold for drought tolerance in alfalfa.

Among the twelve varieties, four (WL298HQ, 30°N, Platu, and WL354HQ) exhibited a significant decrease in germination rate at either 5% or 10% PEG, whereas the remaining eight showed a significant decline only at 15% PEG (P < 0.05), with WL319HQ showing the largest drop (53.27%). This indicates that the most pronounced reductions in germination performance occurred within the 10–15% PEG range.

Regression equations were established using PEG concentration as the independent variable (x) and germination rate as the dependent variable (y), with R² values exceeding 0.82 (**Table 4**). A 50% reduction in germination rate relative to the control was used to determine the critical PEG concentration representing survival thresholds for each variety. Significant differences were observed among varieties in their tolerance to PEG-induced drought stress. WL440HQ exhibited the highest tolerance, with a critical PEG concentration of 18.39%, whereas 30°N showed the lowest tolerance at 15.10%.

**Table 4:** Regression analysis of germination percentage and PEG concentration of different varieties of Alfalfa.

Variety	Y_1_	R^2^	X_1_^50%^
WL363HQ	y=-0.362x^2^+4.338x+86.238	0.899	17.65
WL525HQ	y=-0.362x^2^+4.938x+79.405	0.867	18.12
WL168HQ	y=-0.388x^2^+4.512x+91.595	0.941	17.69
WL298HQ	y=-0.338x^2^+3.829x+89.762	0.866	17.90
WL712	y=-0.29x^2^+3.510x+76.643	0.829	17.39
WL440HQ	y=-0.319x^2^+4.114x+82.214	0.957	18.39
WL358HQ	y=-0.393x^2^+4.274x+84.524	0.978	16.27
WL319HQ	y=-0.350x^2^+2.850x+88.667	0.946	15.34
WL343HQ	y=-0.314x^2^+2.552x+87.452	0.972	15.72
30 N°	y=-0.257x^2^+1.443x+86.810	0.977	15.10
Platu	y=-0.245x^2^+1.621x+84.071	0.990	15.56
WL354HQ	y=-0.057x^2^+-1.324x+93.476	0.954	18.35

Y_1_ is the regression equation between germination rate and PEG concentration of each variety, and R^2^ is the goodness of fit of the quadratic equation (P<0.01); X_1_^50%^ is the PEG concentration (m/v, %) corresponding to a 50% decrease in germination rate.

As shown in **Figure 2**, the germination potential (GE) of different alfalfa varieties varied significantly under different PEG concentrations. Under non-stress conditions (0% PEG), all varieties displayed high GE, particularly WL298HQ and WL168HQ. With increasing PEG concentration, GE generally declined. At 10% PEG, WL525HQ and WL319HQ still maintained high GE (91.67%–94.17%). By 15% PEG, GE became more differentiated: WL525HQ, MXWL003, and WL319HQ experienced sharp declines (8%–40.83%), while WL298HQ and WL168HQ retained relatively high GE (76.67%–80.83%). At 20% PEG, GE across all varieties fell below 44.17%, though WL354HQ performed significantly better than others (44.17% vs. generally <25%). Overall, there was substantial variation in drought tolerance among the tested varieties, with germination potential generally decreasing as drought intensity increased.

The results showed significant differences in germination indices among the 12 alfalfa varieties under non-drought conditions (**Figure 3**). WL168HQ exhibited the highest germination index (89.94), followed by WL525HQ (87.58), WL298HQ (84.25), and 30°N (81.38), while WL358HQ had the lowest value (70.04). Under PEG-6000-induced drought stress, the germination indices of all varieties decreased in a dose-dependent manner. As PEG concentration increased, both germination potential and germination index declined synchronously, with all values dropping below 21 at 20% PEG.

Under mild drought stress, some varieties (WL363HQ, WL298HQ, and six others) exhibited stress-stimulating effects, where their vigor indices exceeded those of the control group. Among them, WL525HQ maintained the highest vigor under both control (4047.7) and moderate stress conditions (10–15% PEG: 3031.07–1248.85). Platu showed the best performance at 5% PEG (4311.78), and WL440HQ performed best under severe stress (20% PEG: 331.78). These findings indicate substantial variation in drought response among the tested varieties, with WL525HQ demonstrating superior performance across most stress levels, while certain varieties exhibited enhanced adaptation under mild stress.

The vigor index (VI) of all varieties decreased significantly with increasing PEG concentration, although distinct response patterns were observed among different genotypes (**Figure 4**). At 5% PEG, several varieties—including WL440HQ, Platu, and WL358HQ—showed VI values higher than the control, suggesting that mild drought may transiently enhance seedling vigor through activation of osmoregulatory mechanisms. However, when PEG concentration reached or exceeded 10%, VI began to decline significantly. At 20% PEG, VI dropped to very low levels across all varieties (e.g., 5.13 for WL343HQ and 3.78 for 30°N), indicating that severe drought exerts a strong inhibitory effect on early seedling growth.

**Figure 5:**
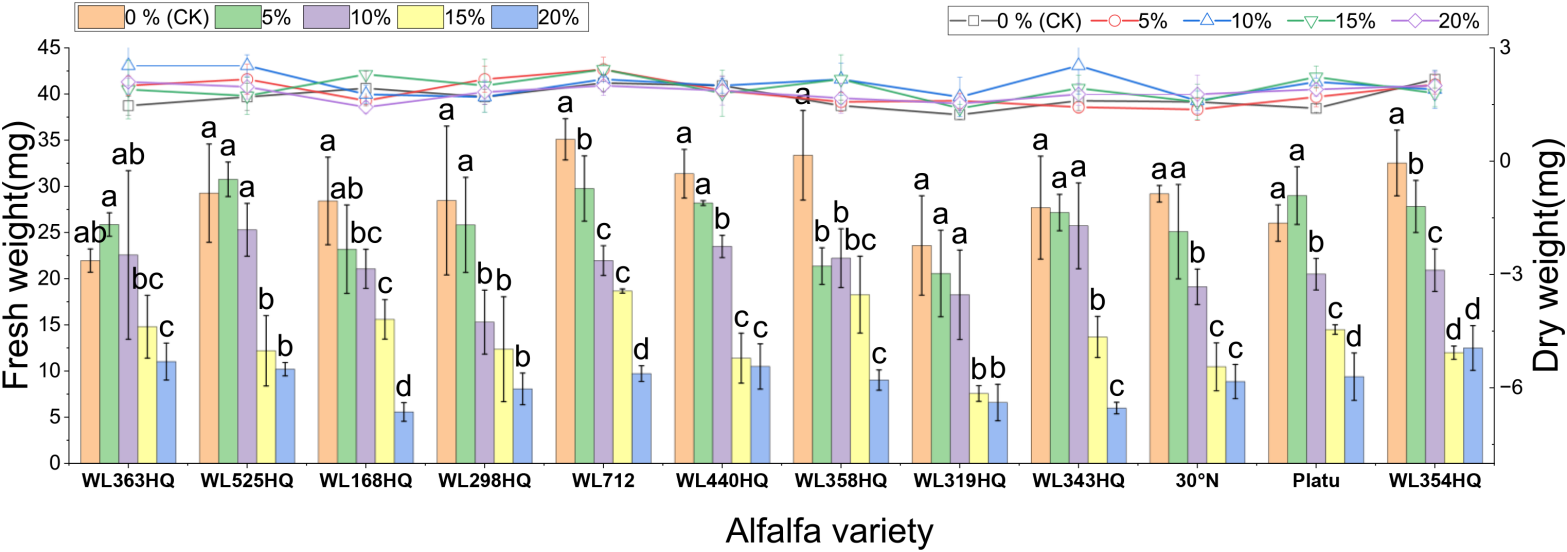
Alfalfa seedling fresh weight and dry weight under drought stress. Different lowercase letters indicate significant differences (P<0.05) in the same index of the same variety of alfalfa under drought stress at different concentrations of PEG, and % indicates different mass concentrations of PEG. The vertical axis represents different varieties of alfalfa. The same applies hereinafter.

**Figure 6:**
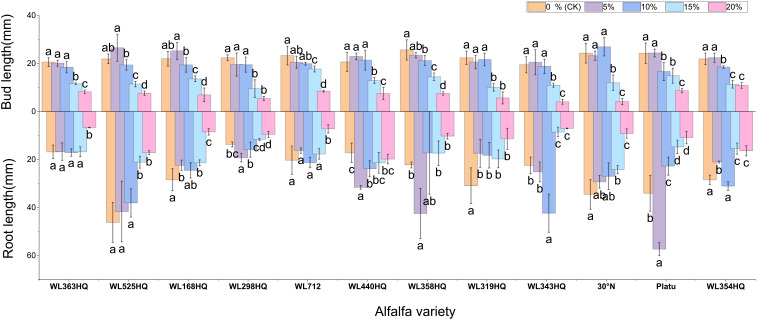
Bud length and root length of alfalfa seedlings under drought stress. Different lowercase letters indicate significant differences (P<0.05) in the same index of the same variety of alfalfa under drought stress at different concentrations of PEG, and % indicates different mass concentrations of PEG. The vertical axis represents different varieties of alfalfa. The same applies hereinafter.

As PEG concentration increased, root length, shoot length (**Figure 5**), fresh weight, dry weight, and relative water content (**Figure 6**) of all varieties decreased significantly, demonstrating the overall inhibitory effect of PEG-induced drought stress on alfalfa growth. In most varieties, root and shoot lengths were longest under control conditions and progressively shortened with increasing stress intensity. WL525HQ had the longest root length under control (46.16mm), which was significantly reduced to 17.11mm at 20% PEG (P < 0.05). WL298HQ had the shortest root length under control (13.66mm), which further decreased to 9.51mm at 20% PEG. Shoot length followed a similar trend, confirming that PEG stress strongly suppresses root and shoot elongation.

Varietal responses in root-to-shoot length ratio varied across PEG concentrations. Some varieties (e.g., WL363HQ, WL712) showed a slight increase at low PEG levels (5%) before declining, whereas others (e.g., WL525HQ, WL358HQ) exhibited increased ratios at high PEG concentrations (15–20%), highlighting genotype-specific responses to drought stress.

Both fresh and dry weights decreased with increasing PEG concentration, indicating reduced biomass accumulation under drought stress. WL363HQ had the highest fresh weight under control conditions (21.97 mg), which dropped significantly to 11.03 mg at 20% PEG. Relative water content also declined with increasing PEG concentration, reflecting impaired water balance in stressed plants. The highest relative water content (95.33%) was recorded in WL358HQ under non-stress conditions, with all other varieties maintaining values above 90%. WL363HQ showed the highest relative water content at 0% PEG (93.33%), which significantly decreased to 80.49% at 20% PEG (P < 0. Effect of bicarbonate stress on alfalfa seed germination.

Most germination and phenotypic indices of the twelve alfalfa varieties were significantly influenced by variety, bicarbonate stress, and their interaction. Relative water content did not differ significantly among varieties but was highly significantly affected by bicarbonate stress and the interaction between variety and bicarbonate stress (P < 0.01; **Table 5**). Except for relative water content, all other indices were highly significantly affected by variety, bicarbonate stress, and their interaction (P < 0.01).

**Table 5:** Two-way ANOVA for the effect of bicarbonate stress and variety and their interaction on alfalfa germination.

Variable		Variety		Bicarbonate stress		Variety× Bicarbonate stress	
		F	P	F	P	F	P
Germination indicators	Germination potential	73.77	**	337.994	**	6.803	**
	Germination rate	71.069	**	271.344	**	7.158	**
	Germination index	101.233	**	465.443	**	6.496	**
	Vitality index	132.565	**	1347.662	**	29.594	**
Phenotypic indicators	Germ length	80.235	**	2573.704	**	19.95	**
	Radicle length	100.411	**	1730.866	**	27.246	**
	Radicle length/germ length	56.261	**	891.887	**	16.887	**
	Fresh weight	15.476	**	524.682	**	4.107	**
	Dry weight	10.964	**	46.616	**	4.213	**
	Relative water content	2.191	ns	15797.926	**	2.342	**

** indicates a significant extreme difference (P<0.01), and ns indicates no significant difference (P>0.05).

**Figure 7:**
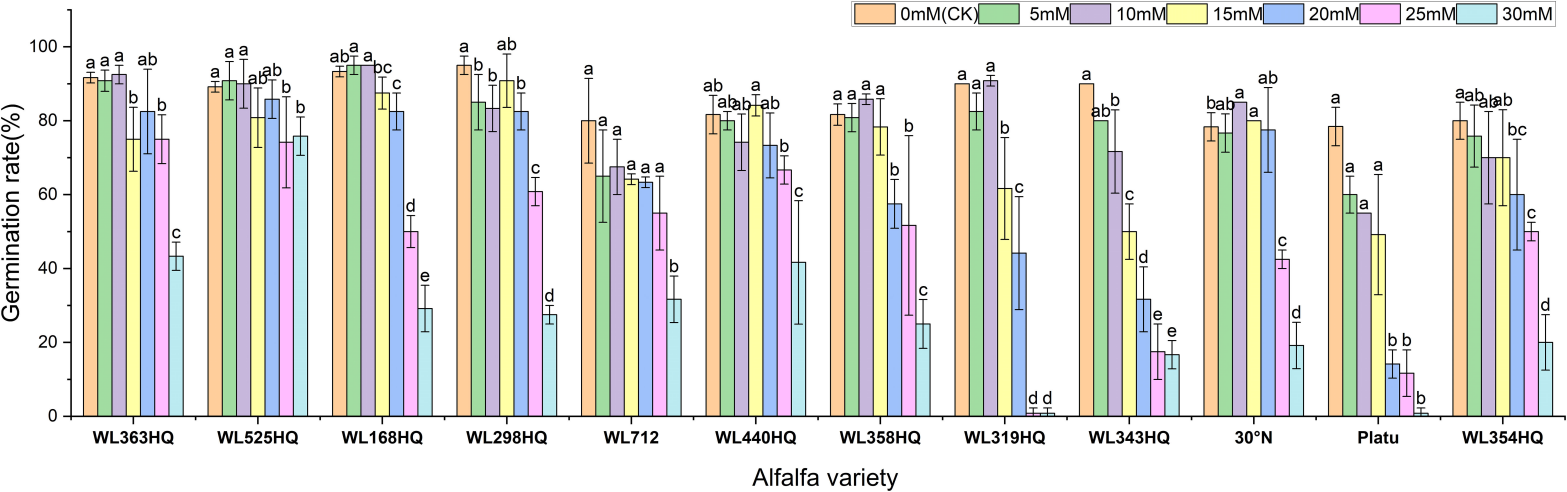
Germination rate of alfalfa seeds under bicarbonate stress. Different lowercase letters indicate significant differences (P<0.05) in the same index of the same variety of alfalfa under drought stress at different concentrations of PEG, and % indicates different mass concentrations of PEG. The vertical axis represents different varieties of alfalfa. The same applies hereinafter.

The overall germination rate of the 12 alfalfa varieties under NaHCO_3_ stress initially increased and then decreased with increasing concentration. As shown in **Figure 7**, the germination rate of each variety responded significantly to NaHCO_3_ concentration (P < 0.01). At low concentrations (0–10 mM), WL168HQ, WL363HQ, and WL525HQ maintained germination rates above 90%, with WL168HQ reaching 95% at 10 mM, indicating strong salinity tolerance. A 5 mM NaHCO_3_ treatment significantly promoted germination in most varieties, with an average germination rate of 79.76%, suggesting that low NaHCO_3_ concentrations may enhance germination through osmotic regulation. However, as the concentration increased (15–30 mM), germination rates declined across all varieties. In particular, WL319HQ and Platu showed drastic reductions (<1%) at 25–30 mM, demonstrating high salinity sensitivity. In contrast, WL525HQ maintained a relatively high germination rate even at 20 mM, indicating notable salinity tolerance.

A regression equation was established using NaHCO_3_ concentration as the independent variable (x) and seed germination rate as the dependent variable (y), resulting in a univariate quadratic regression model for each cultivar after 10 days of stress treatment (**Table 6**). A 50% reduction in germination rate compared to the control was used as the survival threshold, allowing determination of the critical NaHCO_3_ concentration for each variety. These results indicate that different alfalfa varieties exhibit distinct thresholds for salinity tolerance under NaHCO_3_ stress.

**Table 6:** Regression analysis of germination of different alfalfa varieties with NaHCO_3_ concentration.

Variety cultivar	Y_2_	R^2^	X_2_^50%^
WL363HQ	y=-0.071x^2^+0.810x+89.762	0.762	30.05
WL525HQ	y=-0.012x^2^+-0.185x+90.575	0.432	50.95
WL168HQ	y=-0.129x^2^+1.750x+91.607	0.960	25.98
WL298HQ	y=-0.118x^2^+1.750x+87.183	0.864	26.65
WL712	y=-0.091x^2^+1.946x+58.433	0.694	25.08
WL440HQ	y=-0.077x^2^+1.268x+77.798	0.700	28.94
WL358HQ	y=-0.100x^2^+1.167x+80.833	0.828	24.34
WL319HQ	y=-0.094x^2^+-0.589x+92.381	0.899	18.33
WL343HQ	y=0.011x^2^+-3.083x+93.710	0.932	14.98
30 N°	y=-0.174x^2^+3.613x+66.706	0.927	24.66
Platu	y=-0.056x^2^+-0.512x+61.091	0.852	10.23
WL354HQ	y=-0.081x^2^+0.702x+76.607	0.806	22.97

Y_2_ is the regression equation of germination rate and NaHCO_3_concentration for each variety; R^2^ is the goodness of fit of the quadratic equation (P<0.01); X_2_^50%^ NaHCO_3_concentration (mM) corresponding to a 50% decrease in germination rate.

Note: Different lowercase letters indicate that the same index of the same variety of alfalfa was significantly different under different concentrations of NaHCO_3_(P<0.05), and mM indicates different concentrations of NaHCO_3_. The same applies hereinafter.

**Figure 8:**
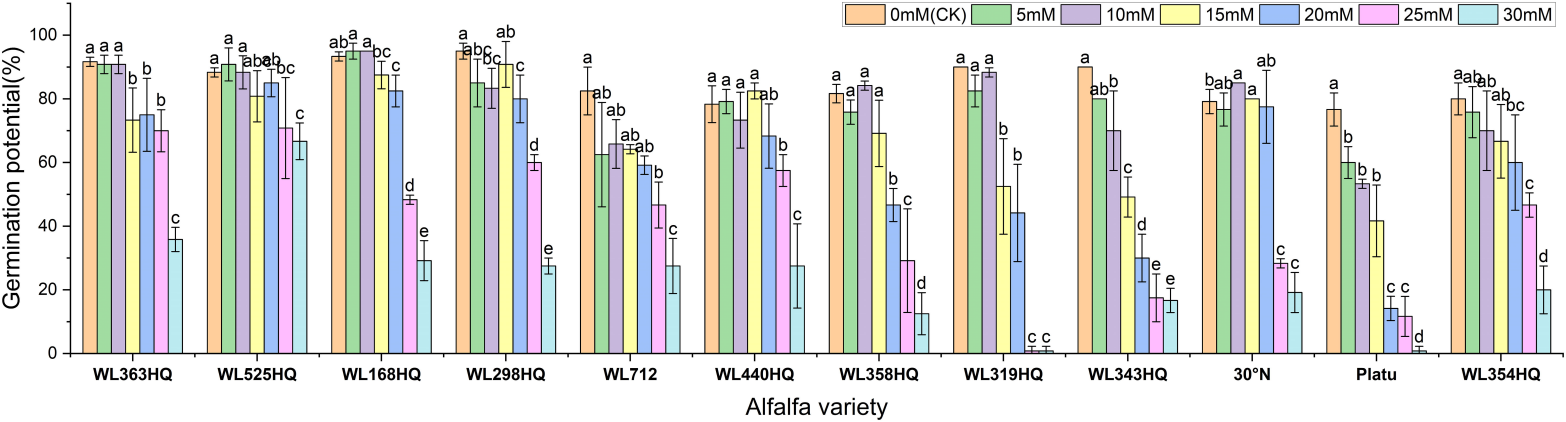
Germination potential of alfalfa seeds under bicarbonate stress. Germination index of alfalfa seeds under bicarbonate stress. Different lowercase letters indicate significant differences (P<0.05) in the same index of the same variety of alfalfa under drought stress at different concentrations of PEG, and % indicates different mass concentrations of PEG. The vertical axis represents different varieties of alfalfa. The same applies hereinafter.

Germination potential analysis further revealed differences in salinity tolerance among varieties (**Figure 8**). WL168HQ maintained a stable germination potential (>90%) at 0–10 mM but showed a significant decline at 15 mM, suggesting its tolerance threshold lies between 10 and 15 mM. WL525HQ exhibited higher germination potential than the control at 5 mM, indicating a promoting effect of low NaHCO_3_ concentration. Similarly, 30°N and Platu showed enhanced germination potential at 5–10 mM, possibly due to active regulatory mechanisms under mild stress. However, at ≥25 mM, germination potential sharply declined across all varieties, with Platu nearly losing its ability to germinate at 30 mM, indicating irreversible damage caused by high NaHCO_3_ levels.

**Figure 9:**
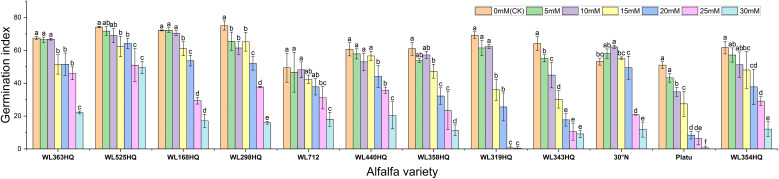
Vigor index of alfalfa seeds under bicarbonate stress. Different lowercase letters indicate significant differences (P<0.05) in the same index of the same variety of alfalfa under drought stress at different concentrations of PEG, and % indicates different mass concentrations of PEG. The vertical axis represents different varieties of alfalfa. The same applies hereinafter.

Based on the response patterns, it was found that 5 mM NaHCO_3_ generally promoted germination, while concentrations >15 mM significantly inhibited it. There was a highly significant difference (P < 0.01) in how germination indices responded to NaHCO_3_ stress among the varieties. As shown in **Figure 9**, seven varieties—including WL363HQ and WL525HQ—showed a steady decline in germination index with increasing NaHCO_3_ concentration, whereas others exhibited fluctuating changes at low concentrations (0–10 mM) before gradually decreasing. WL525HQ demonstrated strong salinity tolerance by maintaining a relatively high germination index even at 25–30 mM. In contrast, WL298HQ, although having the highest germination index (75.13) at 0 mM, showed a sharp decline with increasing stress, indicating high sensitivity. WL168HQ had germination indices of 72.3 and 70.52 at 5 and 10 mM, respectively—the highest values during those stages—while Platu consistently showed the lowest germination index across all concentrations. WL319HQ had a germination index close to zero at high concentrations, indicating very poor salinity tolerance.

**Figure 10:**
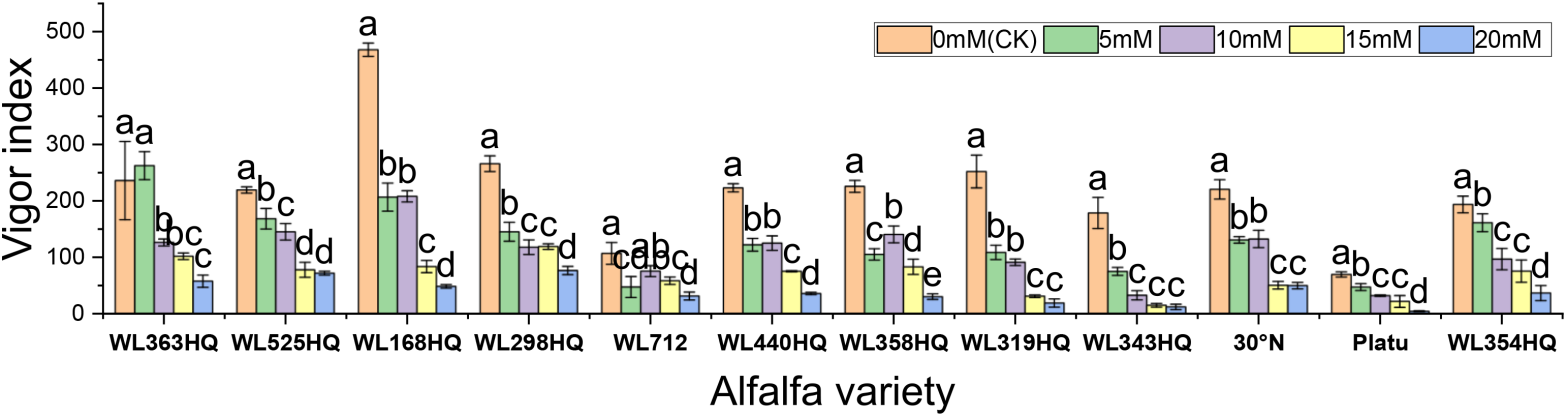
Alfalfa seedling biomass under bicarbonate stress. Different lowercase letters indicate significant differences (P<0.05) in the same index of the same variety of alfalfa under drought stress at different concentrations of PEG, and % indicates different mass concentrations of PEG. The vertical axis represents different varieties of alfalfa. The same applies hereinafter.

As illustrated in **Figure 10**, the germination index of all varieties generally decreased with increasing NaHCO_3_ concentration, though there were clear differences among them. WL363HQ and WL298HQ showed relatively gentle declines under high stress, indicating strong tolerance, whereas WL712 and others exhibited steep declines. WL363HQ reached a vigor index of 2625.15 at 5 mM, which was higher than the control value (2361.47), suggesting that low NaHCO_3_ concentrations may promote growth via osmoregulatory mechanisms. WL168HQ had the highest vigor index (4680.7) under control conditions but declined rapidly with increasing NaHCO_3_ concentration, indicating a narrower adaptive range. During the seed germination stage, different alfalfa varieties exhibited distinct responses to NaHCO_3_ stress. Low concentrations of NaHCO_3_ promoted germination in certain varieties, whereas concentrations above 15 mM exerted significant inhibitory effects. Among the tested varieties, WL525HQ showed a relatively gradual decline in germination index with increasing NaHCO_3_ concentration, indicating stable and superior salinity tolerance.

**Figure 11:**
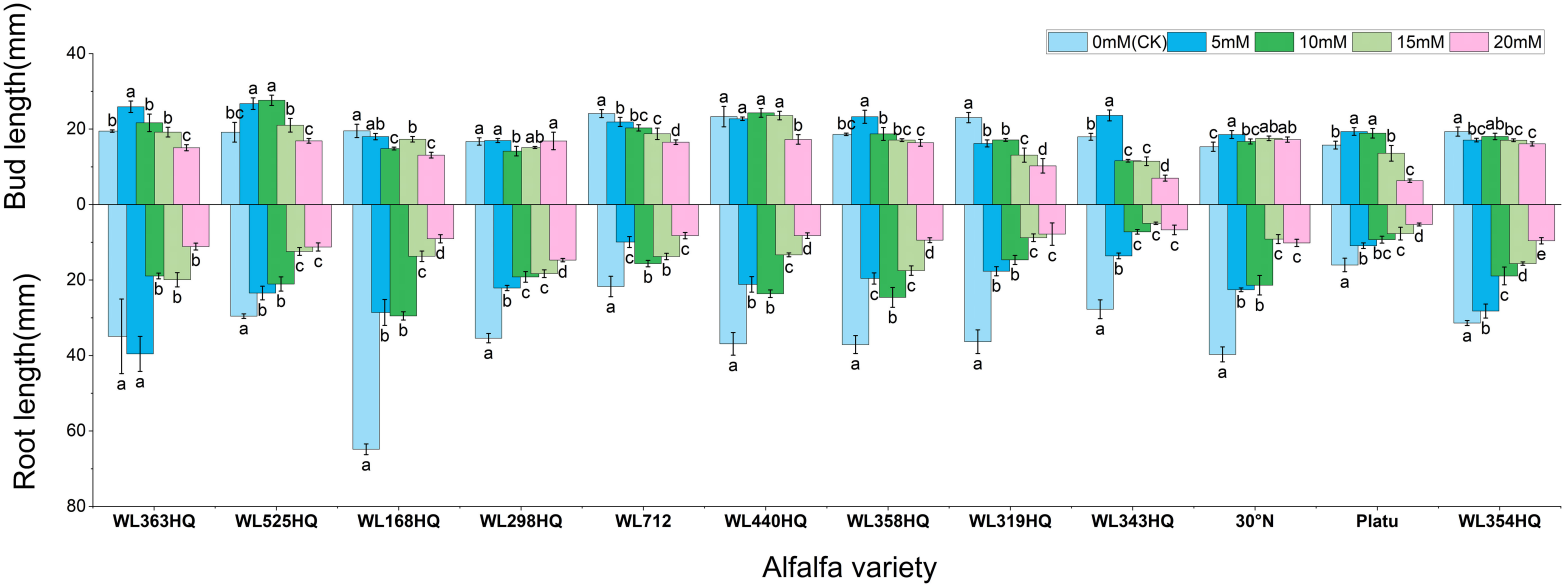
Morphological characteristics of alfalfa seedlings under bicarbonate stress. Different lowercase letters indicate significant differences (P<0.05) in the same index of the same variety of alfalfa under drought stress at different concentrations of PEG, and % indicates different mass concentrations of PEG. The vertical axis represents different varieties of alfalfa. The same applies hereinafter.

As shown in **Figure 11**, different alfalfa varieties exhibited varied responses to NaHCO_3_ stress, with both radicle and shoot lengths being significantly affected (P < 0.05). In the control (CK) group, WL168HQ had the longest radicle length (68.84 mm), while Platu had the shortest (16.01mm); the radicle lengths of other varieties ranged from 20 to 40mm. When NaHCO_3_ concentration increased to 10 mM and above, radicle length generally decreased significantly in most varieties (P < 0.05). Consistent with this trend, most varieties showed a decline in radicle length with increasing NaHCO_3_ concentration. However, WL525HQ reached its maximum radicle length (26.75mm) at 5 mM NaHCO_3_, followed by a gradual decrease with increasing concentration. Although the length declined, the reduction was relatively small compared to the control. At this concentration, WL525HQ also exhibited the longest shoot length (17.25mm).

**Figure 12:**
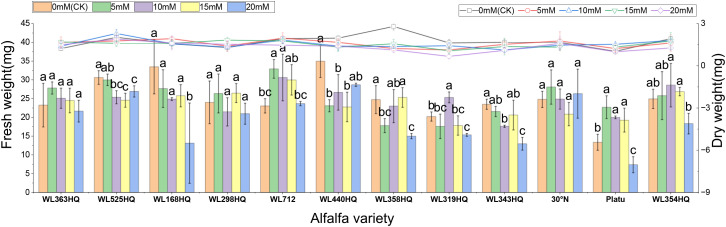
Spectra of 12 alfalfa varieties for drought resistance (1) and salt tolerance (2) cluster analysis during germination period. Different lowercase letters indicate significant differences (P<0.05) in the same index of the same variety of alfalfa under drought stress at different concentrations of PEG, and % indicates different mass concentrations of PEG. The vertical axis represents different varieties of alfalfa. The same applies hereinafter.

Fresh weight generally decreased with increasing NaHCO_3_ concentration in most varieties (**Figure 12**). At low concentrations, some varieties showed little change compared to the control, but significant decreases occurred at higher concentrations (P < 0.05). WL363HQ, WL298HQ, and 30°N showed relatively stable fresh weights across NaHCO_3_ treatments, while other varieties exhibited significant reductions. High NaHCO_3_ concentrations negatively impacted biomass accumulation and water retention. Dry weight followed a similar trend, albeit with smaller reductions compared to fresh weight. Relative water content showed slight fluctuations at low concentrations but declined significantly at ≥15 mM (P < 0.05), reflecting disrupted plant water balance. Varietal sensitivity to NaHCO_3_ varied: WL168HQ experienced a large decrease in relative water content—from 95.31% to 79.49% at 20 mM—whereas WL363HQ remained relatively stable, showing only a 1.6% decrease across all concentrations.

### Cluster analysis

The relative germination potential, relative germination rate, relative germination index, relative vigor index, relative fresh weight, relative dry weight, relative shoot length, relative root length, and relative water content were selected as evaluation indices for assessing the drought resistance and salt tolerance of the twelve alfalfa varieties using the subordinate function method. A higher comprehensive evaluation value indicates stronger drought or salt tolerance.

As shown in **Table 7**, under PEG-6000 treatment, WL440HQ and WL363HQ exhibited the strongest drought tolerance during seed germination, with comprehensive evaluation values of 0.709 and 0.637, respectively. In contrast, 30°N and WL319HQ showed the weakest drought tolerance, with values of 0.171 and 0.182, respectively.

**Table 7:** Membership values and rankings of drought resistance indexes of 12 alfalfa cultivars at germination stage.

Subordinative function value											
Cultivar	RGE	RGP	RGI	RVI	RSL	RRL	RFB	RDB	RWC	Subordinate function value of drought resistant	Ranking
WL440HQ	1.000	1.000	1.000	1.000	0.261	1.000	0.176	0.088	0.857	0.709	1
WL363HQ	0.649	0.516	0.585	0.270	0.767	0.148	1.000	1.000	0.796	0.637	2
WL525HL	0.850	0.850	0.722	0.148	0.990	0.119	0.443	0.610	0.619	0.594	3
WL712	0.552	0.562	0.556	0.211	0.805	0.102	0.126	0.356	0.572	0.427	4
Platu	0.267	0.386	0.388	0.278	0.447	0.059	0.554	0.933	0.454	0.419	5
WL358HQ	0.402	0.307	0.512	0.501	0.334	0.220	0.000	0.707	0.497	0.387	6
WL168HQ	0.558	0.469	0.514	0.135	1.000	0.037	0.141	0.046	0.526	0.381	7
WL354HQ	0.297	0.274	0.273	0.104	0.840	0.348	0.101	0.000	1.000	0.360	8
WL298HQ	0.461	0.383	0.607	0.378	0.000	0.485	0.030	0.427	0.186	0.329	9
WL343HQ	0.147	0.000	0.236	0.345	0.674	0.056	0.395	0.544	0.127	0.280	10
WL319HQ	0.080	0.022	0.346	0.000	0.318	0.115	0.097	0.660	0.000	0.182	11
30 N°	0.000	0.038	0.000	0.059	0.569	0.000	0.042	0.201	0.631	0.171	12

As presented in **Table 8**, under NaHCO_3_ stress, WL712 and WL363HQ demonstrated the highest salt tolerance, with comprehensive evaluation values of 0.787 and 0.782, respectively. Conversely, WL319HQ and WL343HQ were the least salt-tolerant varieties, with values of 0.200 and 0.137, respectively.

**Table 8:** Membership values and rankings of salt tolerance indexes of 12 alfalfa cultivars at germination stage.

Subordinative function value											
Cultivar	RGE	RGP	RGI	RVI	RSL	RRL	RFB	RDB	RWC	Subordinate function Value of salt resistant	Ranking
WL712	1.000	0.902	0.982	0.895	0.320	0.733	0.957	0.548	0.749	0.787	1
WL363HQ	0.726	0.721	0.809	1.000	0.740	1.000	0.622	1.000	0.419	0.782	2
WL525HQ	0.929	1.000	1.000	0.866	1.000	0.814	0.309	0.573	0.464	0.773	3
30°N	0.903	0.936	0.949	0.617	0.896	0.303	0.534	0.562	0.573	0.697	4
WL298HQ	0.554	0.617	0.577	0.619	0.557	0.666	0.505	0.871	0.415	0.598	5
WL354HQ	0.481	0.518	0.513	0.738	0.455	0.814	0.515	0.455	0.632	0.569	6
WL440HQ	0.774	0.801	0.768	0.543	0.556	0.449	0.067	0.328	0.500	0.532	7
WL358HQ	0.595	0.379	0.459	0.533	0.677	0.535	0.224	0.000	1.000	0.489	8
Platu	0.141	0.110	0.066	0.485	0.522	0.646	1.000	0.951	0.450	0.486	9
WL168HQ	0.618	0.695	0.682	0.263	0.331	0.054	0.000	0.747	0.000	0.377	10
WL319HQ	0.053	0.025	0.033	0.151	0.000	0.125	0.420	0.233	0.756	0.200	11
WL343HQ	0.000	0.000	0.000	0.000	0.225	0.000	0.150	0.368	0.495	0.137	12

According to **Table 9**, based on the comprehensive evaluation of drought and salt tolerance among the 12 alfalfa varieties, the ranking from strongest to weakest combined tolerance was as follows: WL363HQ > WL525HQ > WL440HQ > WL168HQ > WL343HQ > WL319HQ > WL712 > WL354HQ > WL298HQ > Platu > WL358HQ > 30°N.

**Table 9:** Comprehensive evaluation of drought resistance and salt tolerance of 12 alfalfa cultivars.

Cultivar	Comprehensive value of drought resistance affiliated functions	Comprehensive value of salt resistance affiliated functions	Comprehensive subordinate function value	Comprehensive resilience ranking
WL363HQ	0.637	0.782	0.709	1
WL525HQ	0.594	0.773	0.684	2
WL440HQ	0.709	0.532	0.620	3
WL712	0.427	0.787	0.607	4
WL354HQ	0.360	0.569	0.464	5
WL298HQ	0.329	0.598	0.463	6
Platu	0.419	0.486	0.452	7
WL358HQ	0.387	0.489	0.438	8
30°N	0.171	0.697	0.434	9
WL168HQ	0.381	0.377	0.379	10
WL343HQ	0.280	0.137	0.209	11
WL319HQ	0.182	0.200	0.191	12

**Figure 13:**
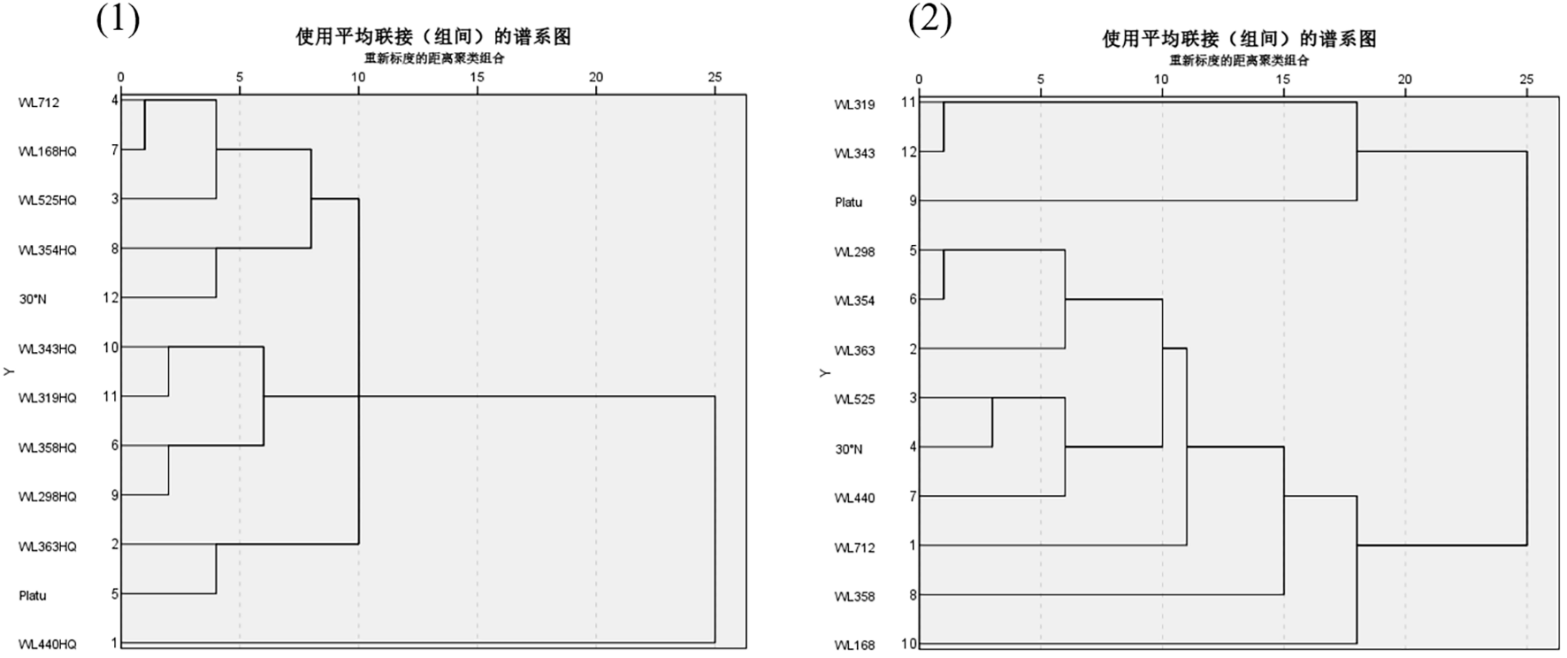
Alfalfa seed germination under combined drought and bicarbonate stresses.

As illustrated in **Figure 13** (1), according to the tree structure, systematic clustering analysis based on Euclidean distance (threshold = 15) classified the drought tolerance of the 12 alfalfa varieties into five categories. The first category included six varieties with strong drought tolerance, accounting for 50% of the tested varieties: WL363HQ, WL440HQ, WL525HQ, WL354HQ, WL298HQ, and 30°N. One variety (WL712) fell into the second category, representing moderate drought tolerance (8.34%). Three varieties—WL358HQ, WL168HQ, and Platu—were grouped into the third category, showing weak drought tolerance (25%). Finally, two varieties—WL319HQ and WL343HQ—were categorized into the fourth group, indicating very weak drought tolerance (16.67%).

As shown in **Figure 13** (2), according to the tree structure, systematic clustering analysis based on Euclidean distance (threshold = 10) divided the salt tolerance of the 12 alfalfa varieties into four categories. The first category consisted of five highly salt-tolerant varieties (41.67%): WL712, WL168HQ, WL525HQ, 30°N, and WL354HQ. The second category included two moderately salt-tolerant varieties (16.67%): WL363HQ and Platu. The third category contained one moderately salt-tolerant variety (8.34%): WL440HQ. The fourth category comprised four weakly salt-tolerant varieties (25%): WL358HQ, WL298HQ, WL319HQ, and WL343HQ.

### Effects of combined drought-bicarbonate on germination of drought-salt-tolerant and drought-salt-sensitive seeds of alfalfa varieties

The drought- and bicarbonate-tolerant variety WL363HQ and the drought- and bicarbonate-sensitive variety WL319HQ were selected for composite stress testing based on an integrated analysis of growth indices from 12 alfalfa varieties under single PEG and NaHCO_3_ treatments during seed germination, using the subordinate function method. The composite stress treatment design is detailed in **Table 2**.

**Figure 14:**
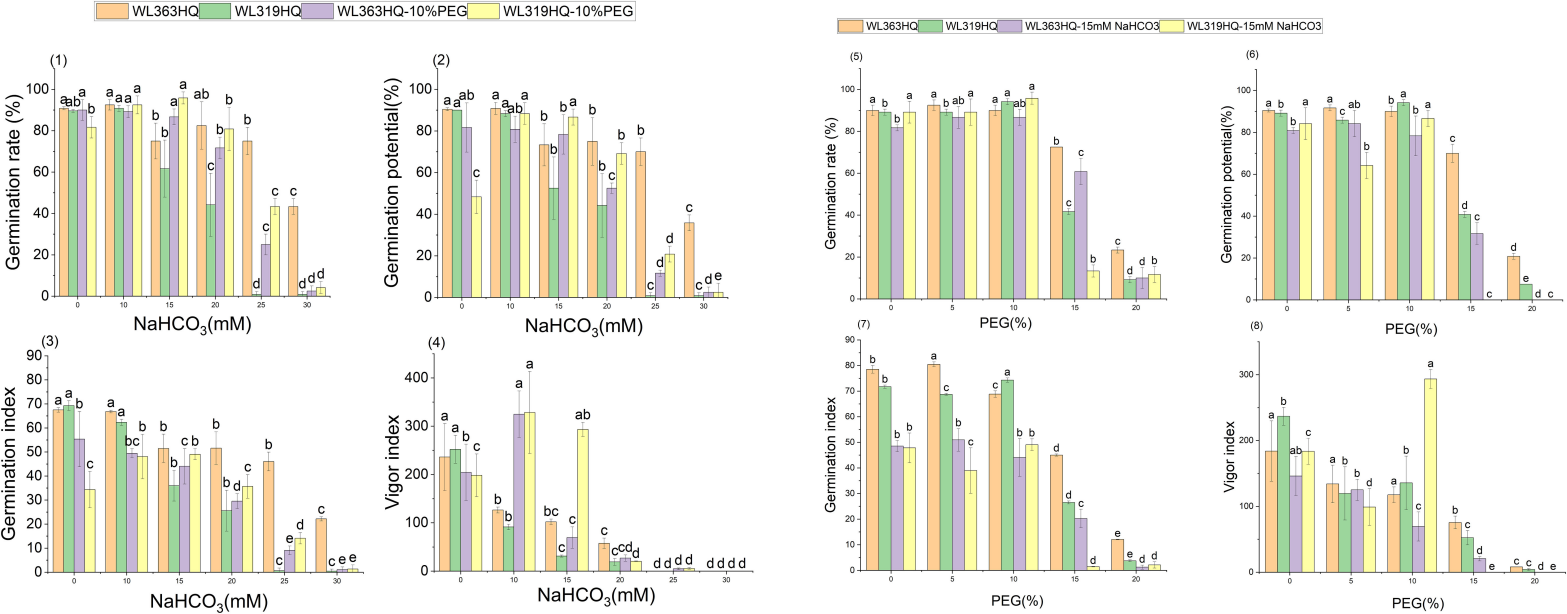
Alfalfa seed germination under combined drought and bicarbonate stresses. Different lowercase letters indicate significant differences (P<0.05) in the same index of the same variety of alfalfa under drought stress at different concentrations of PEG, and % indicates different mass concentrations of PEG. The vertical axis represents different varieties of alfalfa. The same applies hereinafter.

As shown in **Figure 14**-1, group A (fixed 10% PEG with increasing NaHCO_3_ concentrations) and group B (fixed 15 mM NaHCO_3_ with increasing PEG concentrations) significantly affected the germination potential of WL363HQ and WL319HQ. Overall, both varieties exhibited higher germination potential in the low to medium concentration range: 10% PEG + (0–15 mM) NaHCO_3_ and 15 mM NaHCO_3_ + (0–10% PEG), indicating that the inhibitory effects of combined stresses in group B were relatively weaker within this range. In contrast, in the medium to high concentration range—10% PEG + (20–30 mM) NaHCO_3_ and 15 mM NaHCO_3_ + (15–20% PEG)—germination potential was generally higher in group A than in group B, suggesting that increased PEG concentration had a stronger inhibitory effect than NaHCO_3_ stress.

WL363HQ consistently outperformed WL319HQ under both group A and group B treatments, demonstrating superior compound stress tolerance. Notably, under high-stress conditions (10% PEG+30 mM NaHCO_3_ and 15 mM NaHCO_3_ + 20% PEG), WL363HQ showed 2-fold and 1.6-fold higher germination potential than WL319HQ, respectively, highlighting its better adaptation to compound stress. Under group B treatments, WL363HQ maintained relatively stable germination potential (80–90%) at 15 mM NaHCO_3_ + (0–10% PEG), whereas WL319HQ began to decline, further confirming WL363HQ’s stronger buffering capacity against stress.

There were significant differences between the two types of compound stress treatments (P < 0.05), with group B showing lower inhibitory effects than group A. WL363HQ demonstrated better overall tolerance to compound stress compared to WL319HQ, especially under high-stress conditions.

Group A (fixed PEG level) caused significant inhibition of germination in both varieties. Under a fixed 10% PEG background, germination potential decreased from approximately 90% to 20% in WL363HQ and from 85% to 10% in WL319HQ as NaHCO_3_ concentration increased, indicating a clear dose-dependent inhibitory effect. WL363HQ consistently outperformed WL319HQ across all treatment levels. At the highest stress level (10% PEG+30 mM NaHCO_3_), WL363HQ retained about 20% germination potential—twice that of WL319HQ—demonstrating stronger resistance. Even under low-concentration treatments (10% PEG+0–15 mM NaHCO_3_), WL363HQ maintained germination potential within 80–90%, while WL319HQ showed a marked decline. WL363HQ also showed relative stability under moderate stress (10% PEG+15–20 mM NaHCO_3_), with only a gradual decrease in germination potential, suggesting it may possess more effective stress response mechanisms. In contrast, WL319HQ exhibited rapid declines across all treatment levels, indicating high sensitivity to compound stress.

The effects of the two compound stress treatments on seed germination were significantly different (P < 0.05), with clear varietal differences in resistance. Germination rate continuously declined under each concentration gradient in group A. Both varieties showed significant differences in germination index between the two treatments. Group B induced a sharp drop in germination index, particularly under high-stress conditions, indicating strong inhibition by increasing drought stress. WL363HQ displayed a “plateau” response at medium stress levels (15 mM NaHCO_3_ + 10–15% PEG), suggesting activation of adaptive mechanisms such as osmotic regulation or antioxidant defense. WL319HQ, however, showed linear declines across all treatments, implying a lack of effective stress response pathways.

Physiological damage under group B treatments was significantly greater than under group A, possibly due to synergistic effects between NaHCO_3_ and high PEG concentrations. It is hypothesized that NaHCO_3_ may have a detoxifying effect on PEG stress in group A, though this mechanism requires further investigation through transcriptomic analysis to reveal the underlying molecular networks of stress resistance.

**Figures 14**–2 show that group A and group B treatments produced significant differences in vigor indices between WL363HQ and WL319HQ. Overall, vigor index values under group A were generally higher than those under group B, indicating fundamental differences in how the two compound stress types affect plant physiological responses. WL363HQ performed better than WL319HQ in both groups, especially under high-stress conditions (10% PEG+30 mM NaHCO_3_ and 15 mM NaHCO_3_ + 20% PEG), where WL363HQ maintained relatively high physiological activity, while WL319HQ showed significantly reduced performance. This confirms WL363HQ’s stronger compound stress tolerance.

Notably, under group A, both varieties showed decreasing vigor index trends with increasing stress concentration. In contrast, WL363HQ exhibited an adaptive response under medium stress levels in group B (15 mM NaHCO_3_ + 10–15% PEG), maintaining relatively stable index values, which indicates a certain degree of stress buffering capacity.

**Figure 15:**
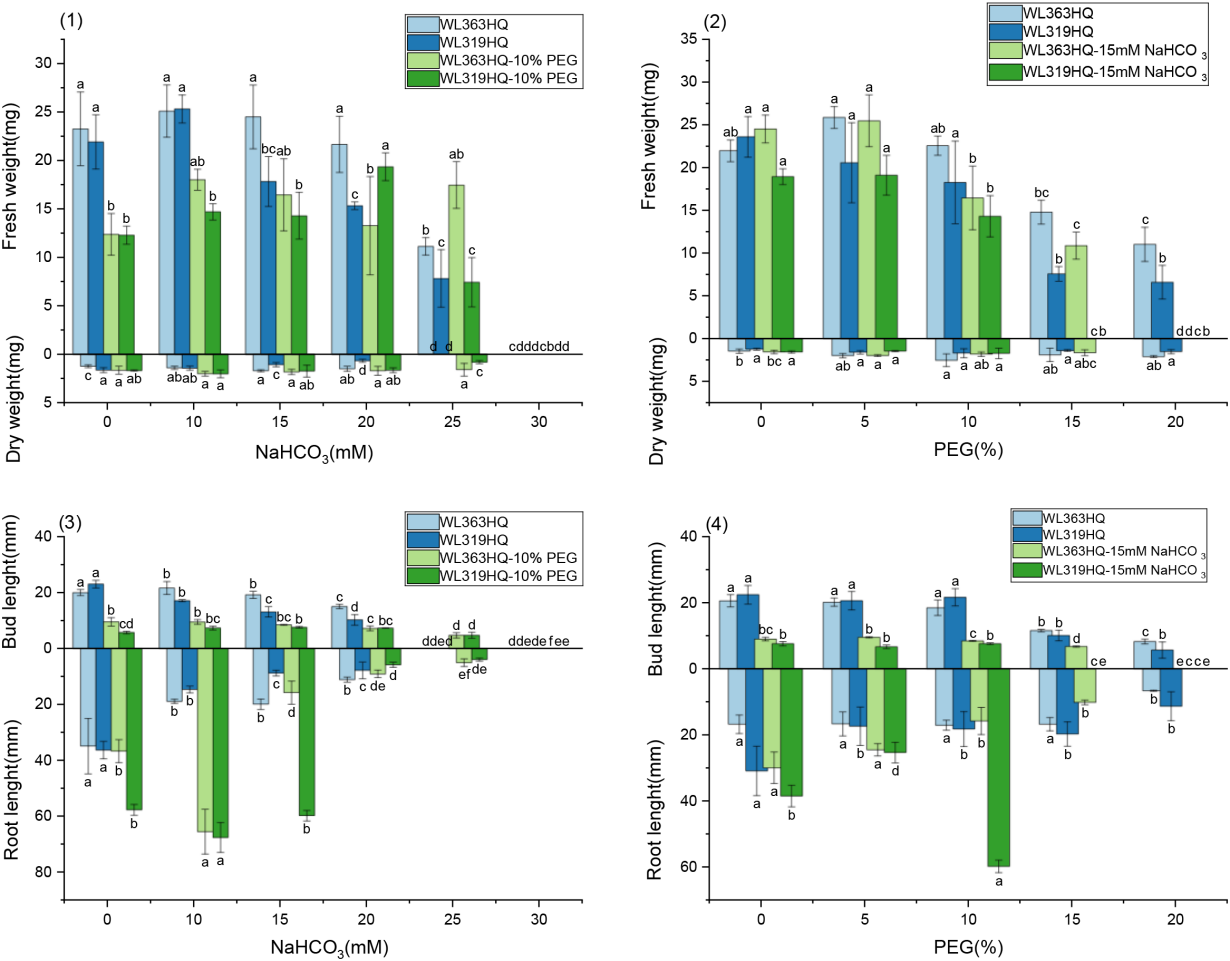
Morphological characteristics of alfalfa seedlings under combined drought and bicarbonate stresses. Different lowercase letters indicate significant differences (P<0.05) in the same index of the same variety of alfalfa under drought stress at different concentrations of PEG, and % indicates different mass concentrations of PEG. The vertical axis represents different varieties of alfalfa. The same applies hereinafter.

At the early stage of seed germination, seeds absorb water and swell, followed by radicle emergence, which serves as a key site for nutrient and water uptake. Root system development reflects plant resistance and adaptability. As shown in **Figure 15**, under group A treatments, the radicle length of WL363HQ and WL319HQ remained relatively stable except under the highest stress condition (10% PEG+30 mM NaHCO_3_), where no germination occurred. Radicle lengths were around 10mm under most treatments. For example, under 10% PEG+10 mM NaHCO_3_, WL363HQ and WL319HQ had radicle lengths of 65.56mm and 67.61mm, respectively—both longer than the control.

Under group B treatments, WL363HQ and WL319HQ showed little change in radicle length, except under moderate salt and severe drought stress (15 mM NaHCO_3_ + 15–20% PEG), where no germination occurred. WL363HQ showed a gradual decrease in radicle length with increasing PEG concentration, while WL319HQ initially decreased and then increased, reaching a maximum of 59.81mm at B3 before failing to germinate under 15% PEG+15 mM NaHCO_3_. At 10% PEG+15 mM NaHCO_3_ (medium salt and moderate drought stress), WL319HQ had a radicle length 3.8 times longer than WL363HQ, indicating that B3 promoted WL319HQ growth while inhibiting WL363HQ.

As shown in **Figure 15**-(1), fresh weight of WL363HQ and WL319HQ fluctuated with increasing NaHCO_3_ concentration under group A treatments, peaking at 10% PEG+10 mM NaHCO_3_ (18.02 mg) and 10% PEG+20 mM NaHCO_3_ (19.34 mg). No significant difference in fresh weight was observed for WL363HQ under 10% PEG+0–25 mM NaHCO_3_. Dry weight first increased and then decreased, peaking at 10% PEG+10 mM NaHCO_3_.

In group B treatments (**Figure 15**-(2)), fresh weight of both varieties increased and then decreased with increasing PEG concentration, reaching maxima at 15 mM NaHCO_3_ + 5% PEG (25.48 mg for WL363HQ and 19.11 mg for WL319HQ). Dry weight followed a similar trend. Across both treatment groups, WL363HQ consistently showed higher fresh weight than WL319HQ, except under 10% PEG+ 20 mM NaHCO_3_ and non-germinating conditions.

**Figure 16:**
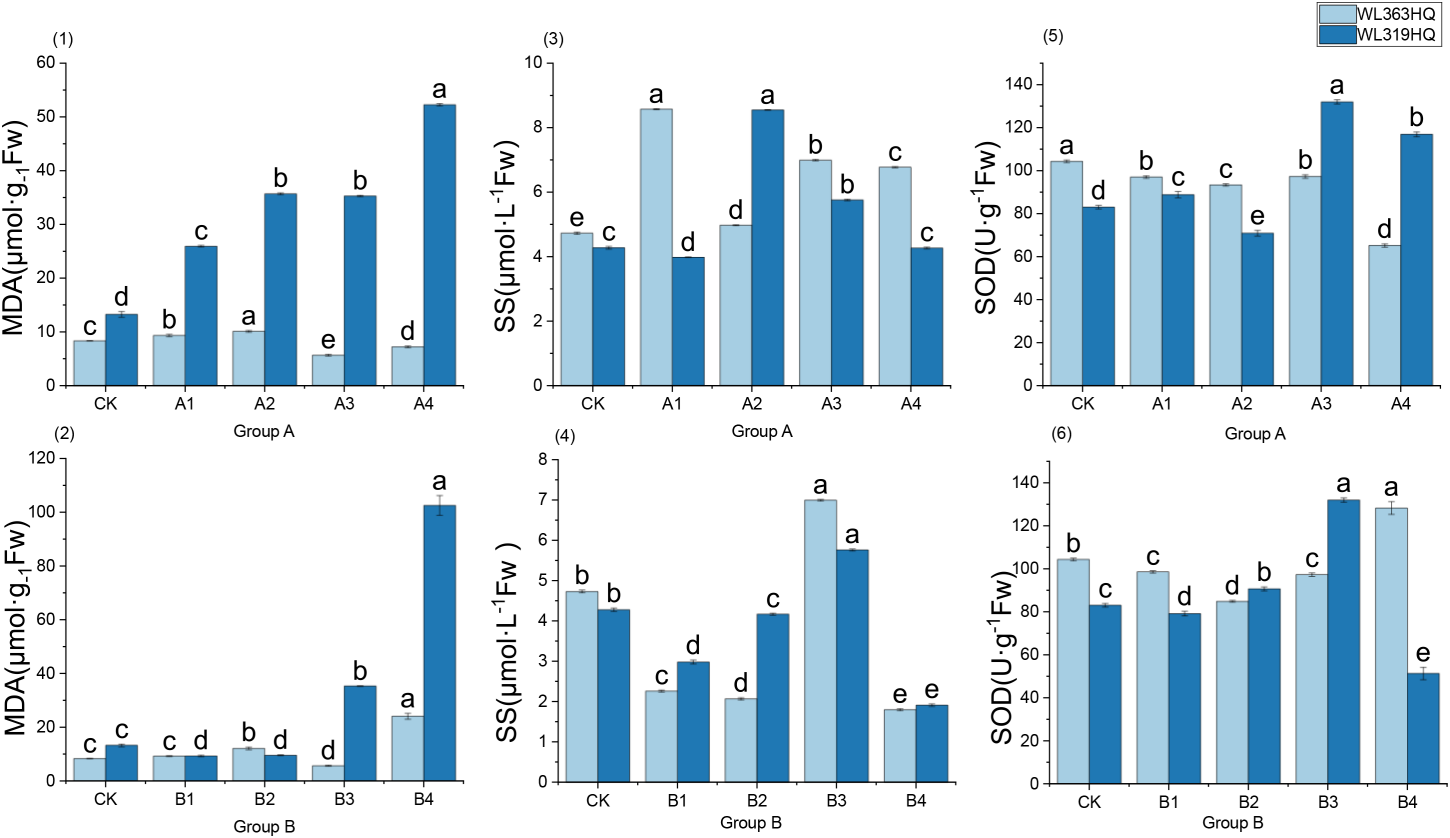
Physiological characteristics of alfalfa seedlings under combined drought and bicarbonate stresses. The horizontal axis of each subplot in **Figure 16** represents the treatment groups under different combinations of drought and bicarbonate stress, with specific proportions referenced in **Table 2**. Different lowercase letters indicate significant differences (P<0.05) in the same index of the same variety of alfalfa under drought stress at different concentrations of PEG, and % indicates different mass concentrations of PEG. The vertical axis represents different varieties of alfalfa. The same applies hereinafter.

From **Figures 16**-(1) and (2), it can be seen that under control conditions, there are significant baseline differences between the two varieties: the MDA baseline of WL363HQ (8.34 μmol mg^-^¹ Fw) is lower than that of WL319HQ (13.27 μmol mg^-^¹ Fw), suggesting a better intrinsic ROS homeostasis. Further coupling MDA with concurrent performance indicators reveals that under A1: 10% PEG treatment, WL363HQ MDA increased by only 1.0 unit, while WL319HQ increased by 12.0 units; under B4 treatment, WL363HQ MDA gradually rose to 24.1 μmol mg^-^¹ Fw, whereas WL319HQ surged to 102.6 μmol mg^-^¹ Fw, with a membrane lipid peroxidation level far exceeding that of WL363HQ. Meanwhile, as NaHCO_3_ concentration increased, germination rates of both varieties declined, but WL363HQ had a slower decrease, ending around 20%, while WL319HQ was only 10%. Both varieties showed initial increases and subsequent decreases in root length, with WL319HQ reaching 59.8mm in B3, 3.8 times that of WL363HQ, indicating a short-term root-promoting effect. Based on this, it is speculated that within 24 hours, ROS surge in WL319HQ exceeded SOD scavenging capacity, leading to inactivation of enzyme metal cofactors (Cu/Zn) and inability to promptly scavenge ·O_2_^-^, ultimately resulting in a sharp increase in MDA and a sudden drop in germination rate; whereas WL363HQ may maintain H_2_O_2_ below 0.18 μmol mg^-^¹ Fw by continuously upregulating antioxidant transcripts downstream of ABA signaling, thereby limiting membrane damage.

As shown in **Figures 16**-(3) and (4), the dynamics of the two varieties of soluble sugars (SS) are synchronized with MDA levels. WL363HQ has a low baseline SS (0.46 μmol L^-^¹) with minimal fluctuations throughout the process; WL319HQ has a higher baseline (0.61 μmol L^-^¹), which sharply rises to 1.94 μmol L^-^¹ under combined stress conditions A4 and B4, exhibiting a stress-induced accumulation pattern of ‘rise then fall.’ Soluble sugars (SS) are not merely ‘osmotic regulators’; their fluctuations may be embedded within an upstream ROS-ABA-SS signaling network. Under moderate to mild combined stress (10% PEG+10 mM NaHCO_3_), SS levels of both varieties increase synchronously, with primary root length reaching a peak (WL363HQ 65.6mm, WL319HQ 67.6mm), indicating a positive correlation between SS accumulation and root apex cell elongation. We speculate that at this stage, SnRK1-mediated sugar signaling activates plasma membrane H^+^-ATPase, maintaining cell wall acidification and relaxation, thereby promoting root elongation.

When stress escalates to B3 (15 mM NaHCO_3_ + 10% PEG), WL319HQ root length remains at 59.8mm (3.8 times that of WL363HQ), yet its SS/MDA ratio has significantly dropped below that of WL363HQ, suggesting that the ‘root length advantage’ may result from temporary osmotic compensation rather than antioxidant benefits. High SS may drive root apex elongation through vacuolar turgor expansion, but continuous ROS damage eventually leads to germination failure under subsequent severe stress (B4). WL363HQ shows an MDA level of 24.1 μmol mg^-^¹ Fw with a germination rate of 55%, whereas WL319HQ exhibits MDA at 102.56 μmol mg^-^¹ Fw with only a 12% germination rate. WL363HQ maintains membrane integrity and relatively high germination under combined stress due to its lower MDA baseline and ‘antioxidant-osmotic coordination’ capability; WL319HQ fails to effectively coordinate ROS scavenging and osmotic regulation, resulting in continued MDA accumulation and performance collapse. This coupled relationship preliminarily supports the ‘antioxidant-osmotic coordination’ hypothesis: high-resistance varieties rapidly accumulate osmolytes (soluble sugars) and limit Na^+^ entry into the hypocotyl, mitigating ROS-induced membrane damage, thereby showing decreased MDA and restored germination rates; sensitive varieties, lacking this coordination, experience synchronized deterioration of MDA levels and performance.

As shown in **Figures 16**-(5) and (6), the SOD activity of the two varieties under combined stress exhibits significant differences: the baseline SOD activity of WL363HQ (104.37 U g^-^¹) is higher than that of WL319HQ (83.06 U g^-^¹), suggesting that the former has a more robust starting point for reactive oxygen species (ROS) scavenging. However, the “absolute value” of SOD cannot be directly equated with stress tolerance; on the contrary, the amplitude and direction of its dynamic fluctuations may better reflect the plasticity of the redox balance. Under the A3 treatment (10% PEG+15 mM NaHCO_3_), the SOD activity of WL319HQ sharply increased to 132.02 U g^-^¹, simultaneously with a rise in MDA and soluble sugars (SS). This phenomenon can be preliminarily interpreted as a “compensatory increase,” but it may also imply the following mechanisms: the inflection point of SOD activity “rising first then falling” may indicate that ROS bursts have exceeded the SOD scavenging threshold, leading to nitration/carbonylation inactivation of the enzyme protein itself; excessive H_2_O_2_, if not timely reduced by APX/CAT, can generate •OH via the Fenton reaction, further exacerbating lipid peroxidation (MDA↑). Alkaline salt (NaHCO_3_)-derived high pH stress may inhibit the metal cofactor binding of Mn-SOD or induce increased protease activity, thereby accelerating SOD turnover; aquaporins (e.g., PIP2;1) are often phosphorylated and closed under alkaline salt conditions, causing faster cellular dehydration, and the chloroplast and mitochondrial electron transport chains become more prone to “electron leaks,” generating additional ROS. The “fluctuation” of SOD activity is not a simple label of sensitivity or tolerance, but a phenotypic output of the 3D network of ROS generation, scavenging, and signaling. Future studies need to integrate transcriptomics (ABA signaling, aquaporins, ion transporters), proteomics (phosphorylation, carbonylation), and real-time ROS imaging to advance the understanding of “antioxidant-osmotic coordination” from mere statistical correlations to causal chains. If the positive correlation between the “SS/MDA ratio” and biomass retention can be validated in larger populations, it may be developed as a rapid biochemical marker of alfalfa stress tolerance under combined stress conditions.

## Discussion

The results demonstrated significant variation among alfalfa varieties in their responses to drought and bicarbonate stress. Under drought stress, WL440HQ and WL363HQ exhibited strong drought tolerance by maintaining high germination rates (>90% at 10% PEG) and long radicle lengths (e.g., WL525HQ with a radicle length of 46.16mm). This aligns with the osmotic adjustment strategy during alfalfa germination reported by previous studies (Ma et al., 2014). Moderately drought-tolerant varieties such as WL712 showed a tolerance threshold of 20% PEG, beyond which germination dropped by more than 50%. Low concentrations of PEG (5–10%) promoted germination in certain varieties like Platu (**Table 4**), potentially due to mild stress-induced activation of aquaporin expression (Zhu, 2016). In contrast, 30°N and WL319HQ experienced over 80% reductions in germination rate at 20% PEG, accompanied by significant decreases in fresh weight and relative water content (P < 0.05), indicating impaired water balance regulation.

Overall, seed germination indices (germination rate, germination potential, etc.) and morphological traits (radicle length, shoot length, etc.) generally declined with increasing PEG-6000 concentration, consistent with findings from previous studies by previous studies (Lu et al., 2023; Wang et al., 2020; Xu et al., 2015).

Responses to bicarbonate stress also varied significantly among varieties. WL525HQ maintained a germination rate above 70% even at 30 mM NaHCO_3_, suggesting its salt tolerance may be linked to mechanisms regulating Na^+^ and HCO_3_^-^ uptake, translocation, and efflux (Ma et al., 2022; Liu et al., 2022; Wu et al., 2019). In contrast, sensitive types such as WL343HQ, WL319HQ, and WL354HQ showed substantial inhibition at 20 mM NaHCO_3_, with germination rates dropping below 60%. Seedlings of all 12 varieties suffered whole-plant necrosis at NaHCO_3_ concentrations exceeding 20 mM, likely due to synergistic effects of osmotic stress, ion toxicity, oxidative damage, and high pH. These stresses ultimately led to energy depletion, membrane system disintegration, and metabolic cessation—processes associated with irreversible damage, as observed in studies by previous studies (Ullah et al., 2022; Gururani et al., 2015; Shi et al., 2025).

The affiliation function value method is widely used for evaluating plant drought tolerance across different varieties or germplasm. Based on this method and nine indicators—including relative germination rate, relative germination potential, relative fresh weight, and relative dry weight—the comprehensive ranking of the 12 alfalfa varieties was determined. WL440HQ ranked highest, indicating the strongest drought tolerance, followed by WL363HQ and WL525HQ. The weakest performers were 30°N and WL319HQ. These findings provide valuable insights for selecting drought-tolerant alfalfa varieties and guiding planting strategies in regions with varying degrees of drought severity.

Drought and bicarbonate stress exerted a dual effect on seed germination—”low promotion and high inhibition.” Germination rates initially increased under low stress levels before declining sharply under higher stress. This biphasic response may result from enhanced osmotic regulation at low stress concentrations, facilitating water absorption and accelerating germination. This pattern aligns with observations from studies by previous studies (Cornacchione and Suarez, 2017; Wan et al., 2025; He et al., 2022; Xiang et al., 2022; Guo et al., 2008).

Alfalfa’s drought and salt tolerance mechanisms are not entirely overlapping. For instance, WL440HQ exhibits strong drought tolerance but only moderate salt tolerance, suggesting divergence in resistance mechanisms. In contrast, WL363HQ performs well under both stresses, implying the presence of cross-resistance mechanisms that warrant further investigation through transcriptomic analysis.

A key finding of this study is the concentration-dependent nature of synergistic and antagonistic interactions between drought and bicarbonate stress. At low concentrations (5–10% PEG+5–15 mM NaHCO_3_), composite stress inhibited WL363HQ germination less than single stress treatments, showing antagonistic effects—possibly due to temporary alleviation of osmotic imbalance by Na^+^. However, at high concentrations (15–20% PEG+25–30 mM NaHCO_3_), WL319HQ germination decreased by an additional 53.2% compared to single stress, indicating synergistic toxicity. This is consistent with findings by previous studies (Nie et al., 2021) in black-fruited *Lycium barbarum*.

As shown in **Table 10**, the physiological basis of this bidirectional interaction may lie in differential energy allocation. Salt-tolerant varieties like WL363HQ maintain ROS homeostasis through elevated SOD activity, whereas sensitive varieties like WL319HQ suffer severe membrane peroxidation under compound stress, with SOD stimulation blocked, leading to antioxidant system collapse and cell death. These findings highlight significant differences in response characteristics and physiological mechanisms between tolerant and sensitive varieties under combined stress. Importantly, this study reveals that “antioxidant-osmotic coordination” is crucial for stress tolerance under compound stress conditions, offering a promising target for future breeding programs aimed at enhancing stress resilience.

**Table 10:** Response characteristics and physiological mechanisms of drought-tolerant and salt-tolerant and sensitive types to compound stress.

Characteristics	Tolerant type (WL363HQ)	Sensitive types (WL319HQ)
Plant length	Longer radicle, shorter radicle	Longer radicle, shorter radicle
Biomass	Fresh weight generally larger	Fresh weight generally smaller
Antioxidant capacity	Stable SOD activity	SOD activity fluctuates
Membrane stability	Low MDA content	High MDA content
Osmoregulation	Gradual accumulation of soluble sugars	Uncontrolled accumulation of soluble sugars

## Conclusion

In this study, we observed significant differences among the twelve alfalfa varieties in their germination responses to drought and bicarbonate stress. Under drought conditions, drought-tolerant varieties such as WL440HQ and WL363HQ exhibited strong adaptability by maintaining high germination rates (>90% at 10% PEG) and long radicle lengths (e.g., WL525HQ with a radicle length of 46.16mm). In contrast, sensitive varieties like 30°N and WL319HQ experienced over 80% reductions in germination at 20% PEG. Similarly, under bicarbonate stress, WL525HQ maintained a germination rate above 70% even at 30 mM NaHCO_3_, while sensitive types such as WL343HQ showed significantly suppressed germination at 20 mM. These findings suggest that genetic background plays a key role in determining stress tolerance, providing a scientific basis for selecting stress-resistant alfalfa varieties.

The interaction between combined stresses exhibited a bidirectional pattern: antagonism at low concentrations and synergy at high concentrations. At low stress levels (5–10% PEG+5–15 mM NaHCO_3_), Na^+^ may temporarily alleviate osmotic imbalance, thereby reducing the inhibitory effect of drought (antagonistic effect). However, at higher concentrations (15–20% PEG+25–30 mM NaHCO_3_), osmotic stress synergized with ionic toxicity, leading to a further 53.2% decrease in germination in the sensitive variety WL319HQ. This non-linear response highlights the importance of considering both stress intensity and varietal characteristics in agricultural practices.

Drought- and salt-tolerant varieties such as WL363HQ demonstrated notable physiological adaptations under compound stress. Their stable SOD activity (104.37 U/g), progressive accumulation of soluble sugars, and low MDA content (8.34 μmol·mg^-^¹ FW) indicate that cellular homeostasis was preserved through enhanced antioxidant defenses and effective osmoregulation. Conversely, the sensitive variety WL319HQ exhibited large fluctuations in SOD activity, uncontrolled accumulation of soluble sugars, and a sharp increase in MDA content (52.27 μmol·mg^-^¹ FW), reflecting oxidative damage and membrane system breakdown. These mechanistic differences offer valuable insights for improving stress tolerance through targeted breeding strategies, such as modulating SOD-related genes or enhancing osmolyte synthesis pathways.

This study systematically analyzed the physiological response patterns and regulatory characteristics of alfalfa during the germination stage under combined drought and salt stress, establishing a scientific evaluation system of ‘single-factor gradient screening—combined stress interaction verification,’ providing a standardized technical framework for research on combined stress in forage crops. From a theoretical perspective, the research results not only fill the gap in understanding the response mechanisms of forage crops to combined drought and salt stress during germination but also enrich the core concepts of plant stress physiology in the following dimensions: first, characteristics exhibited by alfalfa under combined stress, such as “delay in germination threshold and differences in germination recovery after rehydration,” validate the stress memory activation mechanism in plants under abiotic stress, providing empirical support for species-specific and developmental stage-specific studies of stress memory in the field of forage crops; second, the highly resistant variety WL363HQ showed significantly enhanced tolerance to salt stress after drought pre-treatment, directly reflecting the principle of cross-tolerance in plants and further improving the theoretical understanding that ‘single-stress acclimation enhances tolerance to combined stress’; third, addressing the commonly occurring co-existing condition in karst areas of ‘drought aggravating soil salinization,’ this study clearly elucidated response patterns (such as the coordinated changes of osmotic regulators accumulation and antioxidant enzyme activities), providing targeted theoretical support for improving the ecological adaptation of forage in fragile karst regions and filling the research gap on forage adaptation to combined stress in karst areas. From an application perspective, the highly resistant varieties screened in this study (such as WL363HQ) not only provide direct scientific evidence for the deployment of forage varieties in arid and saline-alkali regions but also serve as preferred materials for vegetation restoration and forage cultivation in karst areas, aiding ecological restoration and the coordinated development of the grass-livestock industry in these regions.

Future research needs to further deepen multidimensional exploration: On one hand, it should integrate transcriptomics and epigenomics technologies to analyze the expression regulatory networks of stress memory-related genes (such as WRKY and MYB family transcription factors, and key genes in proline synthesis) in highly resistant varieties, clarify the molecular mechanisms of cross-resistance, and identify key functional genes that can be used in breeding. On the other hand, basic research outcomes need to be translated into practical breeding strategies, specifically including: developing specific molecular markers based on key genes for use in marker-assisted breeding to achieve precise selection of stress-resistant traits and shorten breeding cycles; designing hybrid parent combinations that aggregate ‘resistance genes’ according to stress characteristics in different target regions (arid-saline areas, karst areas), and establishing a complete technical chain of ‘gene function validation — molecular marker development — breeding application’; additionally, integrating UAV-based remote sensing phenotypic monitoring technology to construct a dual breeding standard of ‘molecular markers – phenotypic traits,’ promoting the transformation of alfalfa stress resistance breeding from ‘traditional phenotypic selection’ to ‘coordinated genotypic and phenotypic precision breeding,’ ultimately enabling the research outcomes to deeply empower the sustainable development of the forage industry.

## Data Availability

The original contributions presented in the study are included in the article/supplementary material. Further inquiries can be directed to the corresponding author.
